# Supplementary material for: Harnessing HIV clinics to deliver integrated hypertension care for People living with HIV in Uganda: A formative mixed methods study
Source: PLOS Glob Public Health. 2025 Jun 4;5(6):e0004701. doi: 10.1371/journal.pgph.0004701 (PMC12136366; doi:10.1371/journal.pgph.0004701)
Supplement: S1 Text — (DOCX) [file pgph.0004701.s001.docx]

**S1 KAP Survey Questionnaire**

PLHIV KAP Survey

*PULESA*

*Page 1*

Study No

Level of health facility
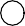
 Tertiary/Referral hospital Tertiary/Referral

Eddaala ly'eddwaliro. hospital


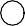
 General/ faith based hospital General/faith based hospital

Health Center IV Health Center IV Health Center III Health Center III


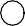

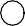


**Social demographic characteristics**

**Ebikwata ku bantu**

1. Age in completed years

Obukulu mu myaka egimaliddwako

1. Gender Female Mukazi

Gender Male Musajja


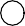

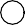


1. What is the estimated distance from your home to the health facility?

Olugendo lwenkana wa okuva mu maka go okutuuka ku (KM) ddwaliro?

1. Education level Never attended school Ssaasoma

Eddaala ly'obuyigirize Primary school Pulayimale Secondary school Sekendule


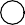

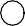

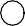

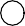


Tertiary/vocational school Tertiary/vocational school

University Univasite


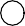

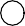

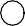


Graduate school

Other Kirala

b.Other,specify Ekirala kirage

1. Occupation? Formally employed Nkozesebwa

Omulimu? Self-employed Nekozesa


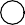

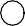

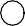

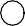

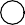


Peasant farmer Ndi mulimi Unemployed Sirina mulimu Others Ebirala

b.Other,specify Ekirala kirage

1. What is your marital status? Married Ndi mufumbo

Oyimiridde otya mu by'obufumbo? Cohabiting Ndi mufumbo atali wa mpeta Single (Never married) Ndi nzekka (siwasanga) Divorced Twayawukana


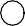

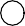

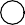

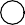

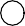

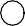


Widowed Namwandu/semwandu Other Ekirala

b.other,specify Ekirala kirage

**Lifestyle risk factors**

**Ensonga z'obuzibu bw'enneeyisa**

1. Current smoker? Yes, I smoke every day Yee,munywa buli lunaku


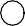

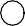


Onywa sigala mu kiseera kino? Yes, I smoke but not every day Yee,naye si buli lunaku


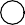
 I do not currently smoke Sinywa sigala mu kiseera kino

1. Past smoker?
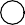
 Yes, I used to smoke every day Yee,nateranga

Wanywako sigala mu kiseera ekiyise? okumunywa buli lunaku


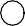
 Yes, I used to smoke but not every day Yee, nateranga okumunywa naye si buli lunaku


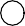
 No, I have never smoked in my life Nedda,simunywangako mu bulamu bwange

1. Do you stay or share a house with someone who smokes Yes Yee cigarretes No Nedda

Obeera oba ogabana ennyumba n'omuntu anywa sigala?


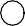

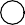


1. How often do you have a drink containing alcohol? Never/Stopped Tekibangawo

Mirundi emeka gy'onywa eky'okunywa ekirimu omwenge? Monthly or Less Buli mwezi oba obutawera


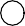

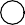

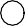

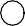

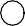


2-4 times a month Emirundi 2-4 mu mwezi 2-3 times a week Emirundi 2-3 mu wiiki

4 or more times a week Emirundi 4 oba okusingawo mu wiiki

1. How many drinks containing alcohol do you have on a 1 or 2 1 oba 2 typical day when you are drinking? 3 or 4 3 oba 4

Byakunywa bimeka ebirimu omwenge byonywa ku lunaku 5 or 6 5 oba 6


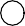

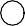

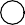

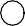

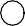


Olwa bulijjo ng'onywedde? 7,8 or 9 7,8 oba 9

10 or more 10 oba okusingawo

1. How often do you have six or more drinks on one Never Tekibangawo

occasion? Less than monthly Obutawera buli mwezi


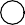

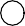

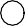

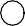


Mirundi emeka gy'onywa eby'okunywa mukaaga oba Monthly Buli mwezi

okusingawo mu lunywa lumu? Weekly Daily or almost daily Buli wiiki buli lunaku oba kyenk buli lunaku

1. What types of alcohol of do you normally consume?

? (Tick all alcohol beverages mentioned by the participant)

Bika ki eby'omwenge by'onywa bulijjo? (Kebera omwenge gwonna ayetabyemu gwayogerako

Local brew Omwenge omuganda Beers Biya

Wines Wayini

Whisky and vodka Whisky ne vodka Others Ebirala

b.Other,specify Ekirala kirage

**Medical and Family history**

**Ebyafaayo by'obujjanjabi ne famire**

1. For how long you have known that you have high blood pressure?

Omaze bbanga ki ng'okimanyi nti olina puleesa? (Months (Emyezi))

1. Do you have any close relatives with high blood Yes Yee?

pressure? No Nedda


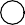

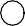


Olina ab'oluganda lwo bonna abalina puleesa?

1. How are you related to the family member with high blood pressure?

Memba wa famire alina puleesa muyita otya?

Mother Maama Father Taata

Siblings (brother or sister) Bato bange (mwanyinaze oba muganda wange Other Ekirala

b.other,specify Ekirala kirage

1. Do you have diabetes mellitus? Yes Yee

Olina obulwadde bwa sukaali? No Nedda


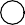

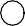


1. Are you taking medication(s) for diabetes Yes Yee

mellitus? No Nedda


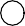

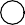


Omira eddagala ly'obulwadde bwa sukaali

1. Are you taking medication(s) for high blood Yes Yee

pressure? No Nedda


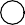

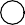


Omira eddagala lya puleesa?

1. For how long have you been taking medications for high blood pressure?

Omaze banga ki ng'omira eddagala lya puleesa? (Months (Emyezi))

1. What medication(s) are you taking for high blood pressure? (Tick all drugs mentioned by the participant)

Ddagala ki erya puleesa ly'omira? (Kebera eddagala lyonna ayeetabyemu ly'ayogera

I don't know the medication(s) Eddagala sirimanyi Nifedipine Nifedipine

Amlodipine Amlodipine Bendroflumethiazide Bendroflumethiazide Hydrochlorothiazide Hydrochlorothiazide Chlorothalidone Chlorothalidone Losartan Losartan

Valsartan Valsartan Telmisartan Telmisartan Captopril Captopril Lisinopril Lisinopril Enalapril Enalapril Bisoprolol Bisoprolol Carvedilol Carvedilol Propranolol Propranolol Other Ekirala

Artenolol

b.Other,specify Ekirala kirage

**Knowledge about high blood pressure**

**Ekimanyiddwa ku puleesa**

1. What is normal systolic blood pressure (top number)?

Enkuba y'omutima eya bulijjo eri ki (nnamba eya waggulu)?

b. What is normal diastolic blood pressure (bottom number)?

Enkuba y'omutima eya wansi eri ki (nnamba eya wansi)?

1. What are some of the risk factors for high blood pressure? Tick all that apply

Nsonga ki eziviirako obuzibu bwa puleesa Kebera byonna ebikola

I don't know any risk factor Simanyi buzibu bwonna bukireeta

Genetics / family related Endagabutonde/bya famire Eating excessive salt Okulya omunnyo omungi

Excessive alcohol consumption Okunywa ennyo omweng Physical inactivity Obutakozesa mubiri

High blood sugar levels Sukaali omungi mu mubiri Overweight/obesity Omugejjo

Excessive body fats Okweyongera kw'amasavu mu mubiri

Stress Okweraliikirira

Chronic kidney diseases Obulwadde bw'ensigo Others, specify Ebirala,birage

b.others,specify Ekirala kirage

1. What are the symptoms of high blood pressure? (tick all symptoms mentioned by the participant)

Obubonero bw'obulwadde bwa puleesa bwe buliwa? (Kebera bwonna ayeetabyemu bw'ayogera )

I donot know simanyi Headache Omutwe oguluma

Pounding heart beat (palpitation) Omutima okukuba ennyo

Difficulty in breathing Okukalubirirwa okussa Dizziness Kamunguluze

High blood pressure has no symptoms Puleesa terina bubonero

Others Ebirala

b.Other,specify Ekirala kirage

1. Can high blood pressure be treated? No Nedda

Puleesa esobola okujjanjabwa? Yes Yee


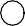

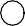

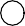


I do not know Simanyi

1. For how long should someone take medications for For life Obulamu bwe bwonna

high blood pressure Only when blood pressure is high/abnormal Nga Omuntu amala bbanga ki ng'amira eddagala lya puleesa puleesa eri waggulu oba nga mbi


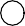

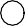


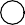
 I do not know Simanyi

1. What were you told your target blood pressure is

? (top number) while on treatment?

Enkuba yo ey'omutima ebeera etya (nnamba eya waggulu)ng'oli ku bujjanjabi?

b. What were you told your target blood pressure is ? (bottom number) while on treatment?

Enkuba yo ey'omutima ebeera etya (nnamba ey'awansi) ng'oli ku bujjanjabi?

1. Does high blood pressure cause damage to body No Nedda

organs? Yes Yee


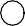

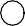

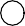


Puleesa ekosa ebitundu by'omubiri? I do not know Simanyi

1. Which organs can be damaged/affected by high blood pressure if not treated well? ( tick all organs

mentioned by the participant)

Bitundu ki ebiyinza okukosebwa puleesa singa tejjanjabibwa bulungi?(Kebera ebitundu byonna ayetabyemu byayogerako)

Brain Obwongo Eye Eriiso Heart Omutima Kidneys Ensigo Others Ebirala

b.Other,specify Ekirala,kirage

**Patients attitudes toward high blood pressure**

**Endowooza y'abalwadde eri puleesa**

1. High blood pressure is not a disease Strongly agree Nzikkiririza ddala

Puleesa si bulwadde Agree Nzikiriza


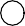

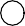

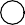


Neither agree nor disagree Sikkiriza ate era sigaana

Disagree Sikkiriza


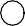

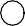


Strongly disagree Sikkiririza ddala

1. High blood pressure is caused by witchcraft Strongly agree Nzikkiririza ddala

Puleesa ereetebwa ddogo Agree Nzikiriza


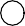

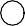

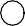


Neither agree nor disagree Sikkiriza ate era sigaana

Disagree Sikkiriza


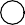

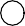


Strongly disagree Sikkiririza ddala

1. High blood pressure only affects rich people Strongly agree Nzikkiririza ddala

Puleesa ekwata bagagga bokka Agree Nzikiriza


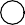

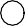

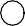


Neither agree nor disagree Sikkiriza ate era sigaana

Disagree Sikkiriza


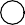

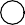


Strongly disagree Sikkiririza ddala

1. High blood pressure only affects people who are Strongly agree Nzikkiririza ddala overweight or obese (very big) Agree Nzikiriza

Puleesa ekwata banene bokka Neither agree nor disagree Sikkiriza ate era sigaana


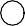

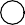

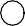


Disagree Sikkiriza


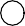

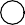


Strongly disagree Sikkiririza ddala

1. High blood pressure always presents with symptoms Strongly agree Nzikkiririza ddala Puleesa blijjo ejjirako obubonero Agree Nzikiriza

Neither agree nor disagree Sikkiriza ate era sigaana


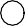

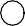

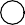


Disagree Sikkiriza


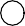

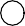


Strongly disagree Sikkiririza ddala

1. High blood pressure can be prevented through Strongly agree Nzikkiririza ddala lifestyle modification, e.g., exercise, reducing Agree Nzikiriza

alcohol intake Neither agree nor disagree Sikkiriza ate era


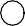

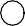

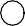


Puleesa esobola okuziyizibwa okuyita mu kukyusa sigaana

enneeyisa, e.g.,duyiro,okukendeeza omwenge Disagree Sikkiriza


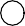

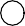


Strongly disagree Sikkiririza ddala

1. High blood pressure can be prevented through Strongly agree Nzikkiririza ddala dietary modifications such as low salt intake and Agree Nzikiriza

eating food with less fat. Neither agree nor disagree Sikkiriza ate era Puleesa esobola okuziyizibwa okuyita mu Kukyusa mu sigaana


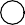

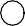

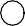


ndya okugeza ng'okulya Omunyo ogw'ekigero n'okulya Disagree Sikkiriza


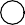

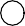


emmere Erimu amasavu amatono. Strongly disagree Sikkiririza ddala

1. Checking blood pressure regularly is important Strongly agree Nzikkiririza ddala Okwekebeza puleesa buli kiseera kikulu Agree Nzikiriza


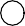

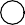


Neither agree nor disagree Sikkiriza ate era sigaana

Disagree Sikkiriza

Strongly disagree Sikkiririza ddala

1. High blood pressure can be treated or cured by Strongly agree Nzikkiririza ddala herbal medicines Agree Nzikiriza

Puleesa esobola okujjanjabibwa oba okuwonyezebwa Neither agree nor disagree Sikkiriza ate era n'eddagala ly'ekinnansi sigaana

Disagree Sikkiriza

Strongly disagree Sikkiririza ddala

1. High blood pressure only affects people with a lot Strongly agree Nzikkiririza ddala of stress Agree Nzikiriza

Puleesa ekwata abantu abalina okweraliikirira okungi Neither agree nor disagree Sikkiriza ate era bokka sigaana

Disagree Sikkiriza

Strongly disagree Sikkiririza ddala

1. I have to take medications for high blood pressure Strongly agree Nzikkiririza ddala daily for life. Agree Nzikiriza

Nina okumira eddagala lya puleesa buli lunaku obulamu Neither agree nor disagree Sikkiriza ate era bwonna. sigaana

Disagree Sikkiriza

Strongly disagree Sikkiririza ddala

1. I can stop my medication as long as I don't feen Strongly agree Nzikkiririza ddala any symptom Agree Nzikiriza

Nsobola okuyimiriza eddagala lyange kasita mba nga Neither agree nor disagree Sikkiriza ate era sikyawulira bubonero bwonna sigaana

Disagree Sikkiriza

Strongly disagree Sikkiririza ddala

**Patients practices towards high blood pressure.**

**Abalwadde bye bakola ku puleesa.**

1. How often do you get your blood pressure measured? Never Tekibangawo

Daily Buli lunaku

Mirundi emeka gy'opimisa puleesa yo? Weekly Buli wiiki Monthly Buli mwezi

Every 3 months Buli myezi 3

Every 6 months Buli myezi 6 Irregular Si bulijjo

1. How often do you take your blood pressure Daily Buli lunaku

medicines? Weekly Buli wiiki

Mirundi emeka gy'omira eddagala lyo erya puleesa? I only take when I feel unwell Ndimira nga

sewulira bulungi Other Ekirala

b.Other,specify Ekirala kirage

1. What is your last blood pressure measurement (top number)?

Puleesa gye wasemba okupimisa yali etya (ennamba eya waggulu)?

b. What is your last blood pressure measurement (bottom number)?

Puleesa gye wasemba okupima yali etya (ennamba eyawansi)?

1. when was your last blood pressure measured? Within 7days Mu nnaku 7

Puleesa yo wasemba ddi okugipimisa? 8-30 days Mu nnaku 8‐30

31-120days Mu nnaku 31‐120

121days and beyond Mu nnaku 121 n'okusukawo

1. Is your blood pressure well controlled ? No Nedda

Puleesa yo ekkakanyizibwa bulungi? Yes Yee

I do not know Simanyi

1. How often do you perform physical exercise? I do not exercise at all Sikola dduyiro n'akamu Otera ddi okukola dduyiro? Regularly Bulijjo

Irregular Si bulijjo

1. How many times do you perform physical exercise in a week ?

Mirundi emeka gy'okola dduyiro mu wiiki?

1. How long do your physical exercises last? Dduyiro wo amala budde ki?

(Minutes (Eddakiika))

1. What kind of physical exercise do you perform ? Mainly sedentary (e.g. sitting, reading, watching Dduyiro okola wa kika ki? television) Okusinga nga ntudde

(e.g.okutuula,okusoma,OkulabaTV)

Mild exercise, minimal effort (eg. yoga, archery, sport fishing, easy walking) Dduyiro atali w'amaanyi (eg. yoga,okukuba akasaale,okuvuba,okutambulako)

Moderate exercise (eg. walking, bicycle riding, or light gardening at least 4 hours per week) Dduyiro omusaamusaamu (eg.okutambula,okuvuga akagaali,okulima okutono nga essaawa 4 buli wiiki)

Strenuous exercise (heart beats rapidly e.g. running/jogging, football, vigourous swimming Dduyiro ow'amaanyi(omutima gukuba nnyo e.g.okudduka,okusamba omupiira,okuwuga)

1. How often do you get assessed for complications I have never been assessed for complications related to high blood pressure? Sikeberwangako buzibu bwonna

Otera ddi okukeberwa ku buzibu obwekuusa ku puleesa? Monthly Buli mwezi

Every three months Buli myezi esatu Every Six months Buli myezi mukaaga Every 12 months Buli myezi 12

I don't know Simanyi

Initials of staff completing the survey

Date survey completed
