# Supplementary material for: Harnessing HIV clinics to deliver integrated hypertension care for People living with HIV in Uganda: A formative mixed methods study
Source: PLOS Glob Public Health. 2025 Jun 4;5(6):e0004701. doi: 10.1371/journal.pgph.0004701 (PMC12136366; doi:10.1371/journal.pgph.0004701)
Supplement: S2 Text — (DOCX) [file pgph.0004701.s002.docx]

**S1 Indepth Interview guide**

**An assessment of current hypertension care (HTN) practices, routines, barriers, and facilitators for people living with HIV at selected health facilities in Kampala and Wakiso Districts in Uganda. A Formative Study PULESA-Uganda**

**In-depth Interview Guide for PLHIV**

**Date: | | / | / | |**

**Interviewer Initials:**

**Health Facility:**

**Introductory Script:**

*Thank you for taking the time to talk with me today. For this formative research, we are trying to learn more about current practice, routines, barriers and facilitators to implementation of evidence-based high blood pressure care in urban and peri-urban HIV clinical settings. This will help us identify and understand the characteristics, interests, behaviors and needs of PLHIV with high blood pressure, their health care providers, health facility managers and policy makers that influence their decisions or actions towards integrated high blood pressure* *-HIV care. Findings from this stakeholder formative study will guide the design of patient-centered interventions for high blood pressure* *-HIV integration and effective implementation strategies for scaling up high blood pressure* -*HIV integrated care in urban and peri-urban HIV clinics in Uganda. There are no right or wrong answers to any of my questions; I would like to learn your opinions about the topics we discuss today. Your answers will be kept private, so no one will be able to know how you personally answered the questions.*

*The information from this interview will help us to learn from you how best to develop strategies to improve screening and treatment of high blood pressure* *in HIV clinics*.

**Background information on the patient**

1. Could you please tell me briefly about yourself, who you are, what you do, where you live and whom you live with?
2. Describe for me what your typical day is like?
3. How long have you had high blood pressure?

**Current practices and routines for *high blood pressure***  **measurement, management and monitoring in HIV clinic setting**

Now I am going to ask you some questions about your experience with high blood pressure

1. Can you tell me how and when you found out that you had high blood pressure?
2. What was that experience like for you?
3. What role does it play in your daily life now?
4. If you had to explain to a friend what high blood pressure is, how would you explain it?
5. What do you think causes high blood pressure?
6. Do you think high blood pressure is preventable? why and why not?
7. Do you think that it is curable? why and why not?
8. What have you done in the past and what are you currently doing to manage your high blood pressure?
9. Can you tell me what medication(s) or other remedies you are taking or have taken for high blood pressure?
10. What role does diet play in managing your high blood pressure, probe how?
11. Does exercise play a role? if so how?
12. How comfortable do you feel about disclosing your diagnosis of high blood pressure with others?
13. How do you monitor your blood pressure at home or outside of home? Why?
14. Who do you trust to give you information or advice on high blood pressure?

**Experiences with high blood pressure measurement, management and monitoring in HIV settings.**

1. How long have you been receiving treatment for high blood pressure in this clinic?
2. Where else do you receive your treatment for high blood pressure?
3. What resources are available in your community for blood pressure (BP) measurement, treatment and monitoring?
4. Tell me about how you access high blood pressure treatment?
5. Please describe for me the processes that you go through to access high blood pressure care in this facility (From the time that you arrive at the facility until you depart probe what happens first, and then, and then ….)
6. What are some of the benefits of accessing high blood pressure treatment within this clinic?
7. What are some of the challenges that you face in accessing care for high blood pressure in this clinic?
8. What recommendations or advice have you been given at this clinic on how to manage your high blood pressure?
9. Tell me about any difficulties you face with accessing high blood pressure medication if at all
10. What difficulties if any do you face taking medication for high blood pressure?

**Perceived need for HTN-HIV integration**

1. What efforts have been made by this clinic to improve high blood pressure care while you have been here?
2. How would you like to see the services for high blood pressure care changed in this clinic if at all? why?

**Perceived priority for HTN-HIV integration**

1. What are your thoughts about receiving high blood pressure care and treatment together with HIV services when you come to this clinic?
2. Based on your experience of accessing HIV services at this clinic, what do you think would be the benefits of this? Why?
3. What do you think would be some of the challenges? Why?
