# Supplementary material for: Harnessing HIV clinics to deliver integrated hypertension care for People living with HIV in Uganda: A formative mixed methods study
Source: PLOS Glob Public Health. 2025 Jun 4;5(6):e0004701. doi: 10.1371/journal.pgph.0004701 (PMC12136366; doi:10.1371/journal.pgph.0004701)
Supplement: S1 Checklist — (DOCX) [file pgph.0004701.s003.docx]

**S1 COREQ (Consolidated criteria for Reporting Qualitative research) Checklist**

| **Topic** | **Item No.** | **Guide questions/Description** | **Reported on Page No.** |
| --- | --- | --- | --- |
| **Domain 1: Research team and reflexivity** |  |  |  |
| *Personal characteristics* | |  |  |
| Interviewer/Facilitator | 1 | Which author/s conducted the interview or focus group | Page 11 |
| Credentials | 2 | What were the researcher’s credentials? E.g. PhD, MD | Page 11 |
| Occupation | 3 | What was the occupation at the time of the study | Page 11 |
| Gender | 4 | Was the researcher male or female? | Page 11 |
| Experience and training | 5 | What experience or training did the researcher have? | Page 11 |
| *Relationship with participants* | | | |
| Relationship established | 6 | Was the relationship established prior to study commencement? | No |
| Participant knowledge of the interviewer | 7 | What did the participants know about the researcher? e.g. personal goals, reasons for doing the research | N/A |
| Interviewer characteristics | 8 | What characteristics were reported about the interviewer/facilitator? E.g. Bias, assumptions, reasons and interests in the research topic | N/A |
| **Domain 2: Study Design** | | | |
| *Theoretical framework* | | | |
| Methodological orientation and Theory | 9 | What methodological orientation was stated to underpin the study? E.g. grounded theory, discourse analysis, ethnography, phenomenology, content analysis | Page 8 |
| *Participant selection* | | |  |
| Sampling | 10 | How were participants selected? E.g. purposive, convenience, consecutive, snowball | Page 9,10 |
| Method of approach | 11 | How were participants approached? E.g. face to face, telephone, mail, email | Pages 9,10 |
| Sample size | 12 | How many participants were in the study? | Pages 10 |
| Non-participation | 13 | How many people refused to participate or dropped out? Reasons? | None |
| *Setting* | |  |  |
| Setting of data collection | 14 | Where was the data collected? E.g. home, clinic, workplace | Page 11 |
| Presence of non-participants | 15 | Was anyone else present besides the participants and researchers? | No |
| Description of sample | 16 | What are the important characteristics of the sample? E.g. demographic data, date | Page 9 |
| *Data collection* | |  |  |
| Interview guide | 17 | Were questions, prompts, guides provided by the authors? Was it pilot tested? | Pages 11 |
| Repeat interviews | 18 | Were repeat interviews carried out? If yes, how many? | N/A |
| Audio/visual recording | 19 | Did the research use audio or visual recording to collect the data? | Page 11 |
| Field notes | 20 | Were field notes made during and/or after the interview or focus group? | No |
| Duration | 21 | What was the duration of the interviews or focus group? | Page 11 |
| Data saturation | 22 | Was data saturation discussed? | Page 11 |
| Transcripts returned | 23 | Were transcripts returned to participants for comment and/or correction? | No |
| **Domain 3: analysis and findings** | | |  |
| *Data analysis* | | | |
| Number of data coders | 24 | How many data coders coded the data? | Pages 12,13 |
| Description of the coding tree | 25 | Did authors provide a description of the coding tree? | Pages 12,13 |
| Derivation of themes | 26 | Were themes identified in advance or derived from the data? | Pages 12,13 |
| Software | 27 | What software, if applicable, was used to manage the data? | Pages 12,13 |
| Participant checking | 28 | Did participants provide feedback on the findings? | No |
| *Reporting* | | | |
| Quotations presented | 29 | Were participant quotations presented to illustrate the themes/findings? Was each quotation identified? e.g. participant number | Yes |
| Data and findings consistent | 30 | Was there consistency between the data presented and the findings? | Yes |
| Clarity of major themes | 31 | Were major themes clearly presented in the findings? | Pages 14-28 |
| Clarity of minor themes | 32 | Is there a description of diverse cases or discussion of minor themes? | Pages 14-28 |
