# Supplementary material for: Harnessing HIV clinics to deliver integrated hypertension care for People living with HIV in Uganda: A formative mixed methods study
Source: PLOS Glob Public Health. 2025 Jun 4;5(6):e0004701. doi: 10.1371/journal.pgph.0004701 (PMC12136366; doi:10.1371/journal.pgph.0004701)
Supplement: Attachment — Submitted filename: Response_to_Reviewers.docx [file pgph.0004701.s006.docx]

Florence Ayebare

Infectious Diseases Research Collaboration

Plot 2C, Nakasero Hill Road,

Kampala, Uganda

25^th^ November 2024

Jepchirchir Kiplagat

Academic Editor

PLOS Global Public Health

Dear Editor,

**Re: Submission ID: [PGPH-D-24-01877] Harnessing HIV clinics to deliver integrated hypertension care for People living with HIV in Uganda: A formative mixed methods study**

We are pleased to resubmit our revised manuscript titled *“Harnessing HIV Clinics to Deliver Integrated Hypertension Care for People Living with HIV in Uganda: A Mixed Methods Study”* for your consideration for publication in *PLOS Global Public Health*.

We sincerely thank the Editorial Board and reviewers for their insightful comments and constructive suggestions. The feedback has been invaluable in enhancing the clarity and rigor of our manuscript. We have carefully addressed each point, and all revisions are highlighted in italics for ease of review.

We appreciate your time and consideration and look forward to your decision.

Thank you for your consideration.

Kind regards,

Florence Ayebare

Corresponding Author

ORCID ID: <https://orcid.org/0000-0002-3343-146X>

**Response to Editor Comments:**

**Journal requirements**

**Comment 1: Address typographic errors noticed in several sections of the document**

**Response:** Thank you for this comment. These have now been addressed.

**Comment 2: Prior to using an abbreviation, spell it out in full. For example, first use of PLHIV in the introduction section is not spelt out in full.**

**Response:**

Thank you for this observation. We have now addressed it on line 77-78. The text now reads as follows:

*“The Uganda Ministry of Health (MOH) 2020 guidelines recommend integrating HIV and non-communicable disease (NCD) care so that for healthcare providers who are treating people living with HIV (PLHIV).”* **(Lines 77 -78, Page 5)**

**Comment 3: Please amend your detailed Financial Disclosure statement. This is published with the article. It must, therefore, be completed in full sentences and contain the exact wording you wish to be published. Please only choose the relevant sentences from below**Please clarify all sources of funding (financial or material support) for your study. List the grants (with grant number) or organizations (with url) that supported your study, including funding received from your institution**

**State the initials, alongside each funding source, of each author to receive each grant**

**State what role the funders took in the study. If the funders had no role in your study, please state: “The funders had no role in study design, data collection and analysis, decision to publish, or preparation of the manuscript.”**

**If any authors received a salary from any of your funders, please state which authors and which funders.**

**Response:** Thank you for your comment. We have revised the financial disclosure statement to read as follows:

*“The National Heart, Lung and Blood Institute (NHLBI) of the National Institutes of Health (https://www.nhlbi.nih.gov) funded this study (UG3HL154501awarded to authors FCS and CTL).* *The funders did not influence the study design, data collection and analysis, decision to publish, or the preparation of the manuscript.”*

**Comment 4: In the online submission form, you indicated, "The datasets used/analyzed during the current study are available from the corresponding author upon reasonable request.”**

**All PLOS journals now require all data underlying the findings described in their manuscript to be freely available to other researchers, either**

1. **In a public repository**
2. **Within the manuscript itself**
3. **Uploaded as supplementary information**

**This policy applies to all data except where public deposition would breach compliance with the protocol approved by your research ethics board. If your data cannot be made publicly available for ethical or legal reasons (e.g public availability would compromise patient privacy), please explain your reasons by return email and your exemption request will be escalated to the editor for approval. Your exemption request will be handled independently and will not hold up the peer review process but will need to be resolved should your manuscript be accepted for publication. One of the editorial team will then be in touch if there are any issues.**

**Response:** Thank you for your comment. We have revised the statement to read as follows;

“*Quantitative data will be made available at Harvard Dataverse (https://dataverse.harvard.edu) once it has been de-identified. Similarly, de-identified qualitative data will be deposited at the Qualitative Data Repository, Syracuse University at https://qdr.syr.edu”*

**Comment 5: Please upload a copy of Figures which you refer to in your manuscript. Or, if the figure is no longer to be included as part of the submission, please remove all reference to it within the text. Please provide separate figure files in .tiff or .eps format.**

**For more information about figure files please see our guidelines:**

[**https://journals.plos.org/globalpublichealth/s/figures**](https://journals.plos.org/globalpublichealth/s/figures)

**https://journals.plos.org/globalpublichealth/s/figures#loc-file-requirements**

**Response:** Figures are now provided in separate .tif files

**Comment 6: We noticed you have uploaded Supporting Information files but have not included a list of legends. Please add a full list of legends for your Supporting Information files after the references list**

**Response:** Thank you for your observation. As suggested, we have now included the list of legends at the end of the reference list **(Lines 674-678, page 37)**

# **Response to Reviewers**

# **Reviewer # 1:**

**Overall**

**Comment 1: An interesting and timely analysis supporting previous findings about barriers to successful implementation of hypertension programming in SSA. What is the integration model used here at the systems and program level? Please describe it in the introduction**

**Response:** Thank you for this observation. We have now described the integration model in the introduction section as follows:

*“The integrated model described in this context refers to the provision of services in a clinic where HIV and hypertension care are offered together. In this model, patients receive both types of care at the same location, from the same healthcare provider. We envision this integration being implemented at the clinic level, allowing patients with hypertension (HTN) and HIV to access services seamlessly.”* ***(Lines 103-111, pages 6 & 7)***

**Comment 2: Often, we say programs are integrated without providing the necessary detail to understand the integrated model. Here, please tell us what systems are integrated (unified supply chain? Does the EMR record both HTN and HIV data? Does the same cadre or health care workers screen and diagnose both diseases? Lab integration?) and what program elements are integrated in this model (coordinated pharmacy pickups for ART and HTN meds? Multi-month dispensing of both types of medications? Differentiated service delivery options for both ART and HTN meds? Same clinic space for both services? Same clinical provider for both services? Same provider gives lifestyle/behavioral counselling? etc) Were integration models for HTN/HIV different at the public and private hospitals that you surveys? How? How might these different models have impacted client survey responses?**

**Response:** The integration model we propose for the intervention involves providing hypertension and HIV services at a single service point, all delivered by the same healthcare provider. This model incorporates integrated health management and information tools. It is designed to leverage the existing HIV platform and infrastructure to deliver effective hypertension care. By using this approach, patients can receive blood pressure screenings, lifestyle counseling, and education, as well as antihypertensive medications during their HIV visits, all in one convenient location.

**Introduction**

**Comment 1: Nice review of relevant literature. Clear explanation of how this current study fits into recent and ongoing pilot studies this group is doing with HIV/HTN integration**

**Response:** Thank you very much for your positive feedback and for recognizing the effort made to review the relevant literature and situate this study within the context of our ongoing studies on HIV/HTN integration. We appreciate your acknowledgment of the clear explanations provided regarding how this study builds on and complements recent work in this area. Your comments affirm the approach we've taken, and we are glad it resonates with the study's goals and context.

**Comment 2: Line 72-73: Reference**

**Response:** The reference below has been added and appears on **Line 74, Page 5** as suggested.

Guwatudde D, Mutungi G, Wesonga R, Kajjura R, Kasule H, Muwonge J, et al. The epidemiology of hypertension in Uganda: findings from national non-communicable diseases risk factor survey. PloS one. 2015;10(9): e0138991

**Comment 3: Line 72: Please explain the difference between hypertension and high blood pressure.**

**Response:** Thank you for bringing this detail to our attention. We used the terms "hypertension" and "high blood pressure" interchangeably; however, there is a difference between the two. Hypertension specifically refers to a sustained, chronic condition of high blood pressure that usually requires medical management or lifestyle intervention and that was what we were referring to. We have made revisions to ensure consistency throughout the document. The revised sentence now reads:

*“Hypertension is a major cause of cardiovascular disease (CVD) disability-adjusted life years (DALYs)…”* ***(Line 72, Page 5)***

**Comment 4: Line 78: “all PLHIV.” Is there an age cut-off at which BP screening should start in these guidelines? Usually age 18 and older – would specify here**

**Response:** The 2020 Uganda consolidated guidelines for the prevention and treatment of HIV do not specify the recommended age for blood pressure (BP) screening. However, this study specifically aimed to assess BP screening practices among individuals living with HIV who are 18 years and older.

**Comment 5: Line 79-80: “PLHIV who are diagnosed with HTN should receive integrated services for both HTN and HIV. What is meant explicitly by integrated services here? Do the Ugandan guidelines define the term integration or a particular integration model(s)? Throughout this paper, the only integration model referred to is integrating NCDs into HIV care.**

**Response:** The Uganda Consolidated Guidelines for the Prevention and Treatment of HIV (2020) recommend the integration of HIV and hypertension services. At the primary healthcare level, these guidelines state that individuals living with HIV should receive education regarding the risk factors for hypertension and undergo screening for hypertension and that those diagnosed with hypertension should receive treatment and prescription refills at the same location where HIV services are provided. We acknowledge that additional services, such as laboratory testing for creatinine and electrolytes or testing for secondary causes of hypertension cannot be fully integrated in our resource limited setting.

To clarify the integration model, we aimed to study in this formative research, we have revised the statement as follows:

*“The 2020 Uganda Ministry of Health (MoH) guidelines, recommend integration of HIV and non-communicable disease (NCD) care, so that people living with HIV (PLHIV) receive both HIV and hypertension care within a single clinical setting, administered by the same healthcare provider, and scheduled on the same day.”* ***(Lines 77-80, page 5)***

**Comment 6: Line 82-83: “However, for successful combined health outcomes for HTN/HIV, it is crucial to follow these guidelines.” This sounds like an advocacy statement. Consider rephrasing to indicate that existing barriers to HTN and HIV integration may limit the efficacy of HTN programs.**

**Response:** Thank you for this observation. We have rephrased it as follows:

*“However, in order to improve clinical outcomes (such as screening and blood pressure control) existing barriers to HTN and HIV integration must be addressed.”* ***(Lines 82-83, page 5)***

**Comment 7: Line 93: Is this work part of the PULESA Uganda study? I would devote a paragraph in the introduction to explaining the PULESA work/findings/integration model to date and how this current study fits in**

**Response:** Thank you for this suggestion; we have included a paragraph explaining the PULESA Uganda study. It reads as follows:

*The* *"Strengthening Blood Pressure Care and Treatment Cascade Metrics Among PLHIV in Peri-Urban and Urban Areas - Implementation Strategies to Save Lives (PULESA-Uganda)" study is an implementation trial aimed at improving hypertension care metrics among people living with HIV (PLHIV) in a sustainable way. This study is one of six projects under the Heart, Lung, and Blood Co-morbidities Implementation Models for People Living with HIV, funded by the United States National Heart, Lung, and Blood Institute. Before conducting the trial, we carried out a formative study to understand the context, needs, attitudes, and practices of PLHIV with hypertension who attend HIV clinics in the Kampala and Wakiso districts****. (lines 112-119, page 7)***

**Comment 8: Line 107: would remove “for example, data collection tools were developed based on the CFIR 2009,” as this is already explained in lines 104-105.**

**Response:** Thank you for this comment. We have removed this statement. The sentence now reads:

"*This study was designed and conducted before the publication of the updated 2022 version of CFIR.”* ***(Lines 126-127, page 7)***

**Comment 9: Line 109: “integrated model”: still unclear what this integrated model entails.**

**Response:** The integrated model presented facilitates the provision of hypertension and HIV services within a "one-stop shop" framework, allowing people living with HIV (PLHIV) to access both services during a single visit. The services integrated include blood pressure measurement for screening and subsequent monitoring of hypertension control counseling on the clinical consequences of hypertension, need for treatment (including lifestyle modification and medication) and monitoring for and mitigating any side-effects of medication, this counseling is provided by the same health care providers and counselors who provide HIV care. Prescription and dispensing of anti-hypertensive medications at the same pharmacy as antiretroviral therapy.

**Comment 10: Line 109-112: “Following analysis, the team agreed that the following 110 constructs were relevant for the study to develop strategies to address patient-level gaps to inform intervention components for patient-centered care in the larger trial.” Consider moving to discussion as it is not relevant in the introduction.**

**Response:** Thank you for this suggestion. We have removed this from the introduction.

**Comment 11: Line 113-114: “We used the CFIR framework to identify implementation determinants to improve HTN care within the HIV program.” Consider moving this to the topic sentence of the paragraph**

**Response:** Thank you for this comment and observation. This change has been effected as guided by the reviewer **lines 124-126, Page 7**).

**Methods**

**Comment 1: Very detailed and well-thought-out section. The study design is clearly explained.**

**Response:** Thank you very much for your kind feedback. We appreciate your recognition of the effort put into detailing the study design. We are glad that this section's clarity and thoroughness have helped you understand our approach to the study.

**Comment 2: Line 121-122: “and perceptions toward HTN in the context of HIV and integrated HIV-HTN care.” This reads awkwardly; consider rephrasing**

**Response:** Thank you for this observation. We have amended the sentence to read as follows:

*“Our goal was to evaluate PLHIV’s knowledge, attitudes and practices regarding HTN care, as well as their perception of an integrated HTN-HIV model.”* ***(Lines 127-128, page 7)***

**Comment 3: Line 128: “Three hospitals” What type of hospitals –national, regional, or district?**

**Response:** Thank you for seeking clarification. One of the hospitals is a national referral hospital, while two are non-government, non-profit faith-based hospitals*.* This has been clarified in ***Lines 144-145, page 8***.

**Comment 4: Please present data on all the clients you identified as eligible. How many decided to enroll?**

**Response:** Thank you for the recommendation. The sentence has been revised and now reads as follows:

*“A total of 537 PLHIV with HTN were identified, of these 439 (81.7%) were successfully contacted and 394 (73.4%) participated in the survey ….”* ***(Lines 242-243, page 14)***

**Comment 5: Line 161: how were the targeted sample sizes for each facility chosen?**

**Response:** Thank you for this feedback. We have provided more detail to the section on “study participants” to explain further how the target sample size for each clinic was obtained.

*“We estimated the number of PLHIV with hypertension at each clinic by applying a hypertension prevalence rate of 20.9%. We obtained the number of PLHIV with hypertension at each clinic and the overall total. We then calculated a proportion for each clinic by dividing the total number of PLHIV with hypertension at each clinic. We applied this proportion to the calculated sample size. This approach ensured that participants were recruited from each clinic in equal proportions. However, the target sample size was not reached in clinics where hypertension screening was not conducted, or documentation was lacking.”* ***(Lines 156-162, page 9)***

**Comment 6: Line 213: Figure 1 is terrific**

**Response:** Thank you for your enthusiastic feedback on Figure 1

**Comment 7: Line 221: Table 3: Distance from home to clinic: please specify the unit of measurement**

**Response:** Thank you for this observation. The unit of measurement is kilometers, and we have included this in Table 3. **(Lines 266 - 267, page 15)**

**Results:**

**Comment 1: Comparing survey results from clients at public vs. private facilities would be interesting as the integration models at these two facilities likely differ; consider adding this analysis**

**Response:** We appreciate your comment. We have now expanded our analysis to compare public and private entities and highlight their differences. The complete results section spans from **Lines 240 -456 on pages 14 to 28.**

**Comment 2: Lines 236-239: Consider clarifying these two sentences, as they seem a bit redundant.**

**Response:** We have clarified the two sentences, which now read as follows*:*

*“The level of awareness among patients regarding hypertension-related concepts was notably low. Only 32% of patients correctly identified the systolic BP threshold of 140 mmHg for diagnosing hypertension (HTN), while 24.1% accurately identified the diastolic BP threshold of 90 mmHg”* ***(Lines 278-280, page 18)***

**Comment 3: Lines 249: “We identified patient needs and resources as a key barrier to the integration of hypertension into HIV care,” I don’t understand the thinking here. Is the ‘patient need’ the need to have blood pressure checked very frequently? This is not consistent with evidence-based guidelines. What is the patient resource identified here? How do these needs (or resources) act as a barrier to integration?**

**Response:** “Patient needs and resources” is one of the constructs from the CFIR framework under the outer setting domain [the extent to which patient needs and the barriers and facilitators to meeting these needs are prioritized] that emerged from patient interviews. We have now rewritten this to ensure clarity. It now reads as follows**:**

*“The study assessed patient needs and the extent to which barriers and facilitators to meeting those needs were recognized and prioritized. Findings from the study revealed that HTN services within HIV clinics were a low priority within health facilities, contributing to gaps in care. Routine blood pressure screening was not being conducted, limiting early detection and management of hypertension and poor access to anti-hypertensive medications presented a significant barrier to patient care.”* ***(Lines 295-300, page 22)***

**Comment 4: Line 254-257: How often should clients have their blood pressure checked to qualify as ‘optimal hypertension care’? It’s unclear to me why this paper suggests that having blood pressure checked within the last seven days constitutes optimal care. I doubt the Ugandan national guidelines recommend blood pressure checks this frequently for hypertensive clients. Do these clients have home blood pressure cuffs?**

**Response:** Thank you for highlighting this. Indeed, to make a diagnosis of hypertension at home, guidelines recommend two measurements per day for a minimum of three and ideally 7 days, with an average of the measurements being used to diagnose hypertension. Indeed, guidelines do not specify how frequently someone with hypertension should be checking their blood pressure at home, this is usually tailored to the individual patient’s situation. Given the limitations of blood pressure monitoring in our setting, we used BP checks within seven days as one proxy indicator for optimal care.

**Comment 5: Line 264: It would help to have some background on the integration models in these various care settings; for example, some ‘integrated’ models have hypertension screening in the ART clinic but then refer to NCD clinics or OPD for hypertension treatment. This client’s report of outside referrals for HTN care could be a critique of the integration model itself rather than a critique that the HIV clinic is providing inadequate services.**

**Response:** Thank you for this comment. Our definition of integrated HIV-HTN care was that PLHIV with comorbid hypertension would receive services for both conditions at the same clinic, provided to the same provider, and at the same time. The findings from this formative study indicate that although the guidelines recommended that patients with HIV and HTN services should receive these services from the same point, some clients would receive fragmented services. We document instances where some clients received HIV services in the HIV clinic and received hypertension care from another clinic within the same facility or outside the facility. This indicated partial integration of HIV-Hypertension services.

**Comment 6: Line 271: ‘Exorbitant’ is subjective: would you provide actual prices and show (rather than tell) that these prices are very high, or would state ‘private clinics allegedly sold these medications at prices that caused financial difficulties for patients.”**

**Response:** Thank you for this comment. We have amended the sentence to read as follows:

*“However, patients reported that private clinics often charged higher prices than they could afford for these medications, which created financial hardship; as one participant described…”* ***(Lines 332-333, page 22****)*

**Comment 7: Line 315: “Wakiso district had a significantly higher score of 28.6 compared to Kampala district with a score of 26.1”. Was this difference statistically significant? If so, please provide a p-value. If not, remove the modifier ‘significantly.’**

**Response:** Thank you for this comment. We have removed this table to avoid confusion.

**Comment 8: Line 317: same comment for ‘significantly.’**

**Response:** Thank you for this comment. We have removed this table to avoid confusion.

**Comment 9: Line 318: Figure 3: I cannot tell which box plot corresponds to which score. Please label the figure more clearly and label the Y axes. Only one of these 4 box plots has significant P values – which score does this correspond to?**

**Response:** In order to improve clarity, figure 3 has been removed.

**Discussion**

**Comment 1: Line 374: “These findings suggest that strengthening and integrating HTN care services is critical to overcoming this barrier.” It seems that integration is the only answer to the patient-level concerns you have identified. For example, ‘integration’ per se will not ensure that blood pressure medications are free or well-stocked at the HIV pharmacy. ‘Integration’ does not ensure that clinical staff is well-trained in hypertension counselling, screening and treatment. You need alternate funding models (increased government or donor support? National health insurance fund? Revolving fund pharmacies? etc), supply chain support for antihypertensives, increased support for HRH through training and task-shifting, hypertension screening and education in the community (i.e. not only facility-based) etc. Is hypertension treatment streamlined with simplified treatment algorithms of a few antihypertensive medications and policies that permit task-shifting and nurse-prescribing? Overcoming these barriers is more complex that ‘strengthening integration’. Please be more nuanced about your recommendations. It is not accurate to suggest that an unspecified model of ‘integration’ will fix these barriers**

**Response:** Thank you for this very insightful comment. We have expanded the discussion section to clarify the model recommended **(Lines 458 -539, pages 29-32)**

**Comment 2: Line 431-432: reference**

**Response:** Thank you for this. We have now added these two references below:

Kwarisiima D, Atukunda M, Owaraganise A, Chamie G, Clark T, Kabami J, et al. Hypertension control in integrated HIV and chronic disease clinics in Uganda in the SEARCH study. BMC public health. 2019; 19:1-10.

Heller DJ, Balzer LB, Kazi D, Charlebois ED, Kwarisiima D, Mwangwa F, et al. Hypertension testing and treatment in Uganda and Kenya through the SEARCH study: An implementation fidelity and outcome evaluation. PloS one. 2020;15(1):e0222801.

**Conclusion:**

**Comment 1: Line 437-438: “To address these gaps, health education for co-morbidities with HIV should be routinely integrated into HIV care.” This is a bit sparse for a conclusion. Perhaps circle back to how this study fits into the larger PULESA framework and what future and ongoing work consists of**

**Response:** Thank you for this comment. We have endeavored to expand our conclusion. It now reads as follows:

*“Integrating hypertension services into HIV clinics in Uganda offers an opportunity to address key barriers, including knowledge gaps, inconsistent medication access, and fragmented care delivery. Addressing these issues will require collaboration between policymakers, healthcare providers, and community stakeholders to create a cohesive, patient-centered approach that supports dual management of HIV and HTN within resource-constrained environments. Furthermore, these insights underscore the importance of incorporating health education on comorbidities into routine HIV care to foster patient understanding and engagement. This study’s findings informed targeted strategies to integrate HTN care into HIV settings, focusing on patient-specific barriers such as knowledge deficits, financial constraints, and service misalignment”* ***(Lines 555-563 Page 33)***

**Reviewer # 2:**

**Comment 1: This paper has the potential to make an important contribution in the area of overlap in the need for care among adults/older adults living with HIV and hypertension (or other NCDs) in sub-Saharan Africa. The front end of the paper is very well written and clearly outlines the issues that need attention. The paper makes a unique and rich data source from Uganda that includes both survey data and qualitative interviews with individuals seeking care for HIV, who also have HTN (or are at risk of HTN). As is clearly pointed out, despite there being a policy for HIV-NCD integration, the reality on the ground is that this is not the experience that most patients in these hospitals/clinics experience. Using a well-established framework, Consolidated Framework for Implementation Research (CFIR), the authors aim to offer the experience of patients in these settings and use these findings to outline recommendations**

**Response:** Thank you for this comment.

**Comment 2: The authors allude to the benefits of their mixed methods design. Similar designs have been called – data-linked nested studies – and some ways to take advantage of the unique added value of the data are outlined here: Schatz E. 2012. Rationale and procedures for nesting semi-structured interviews in surveys or censuses. Population Studies 66(2): 183-95.** [**https://doi.org/10.1080/00324728.2012.658851**](https://doi.org/10.1080/00324728.2012.658851)**.**

**Response:** Thank you for sharing this paper. Our study employed a parallel convergent mixed methods design, where both quantitative and qualitative approaches worked together to address the research question. Specifically, our qualitative sample was drawn from the larger survey sample. We purposefully selected eligible participants from this broader survey group.

**Comment 3: The findings and recommendations align well with other work from sub-Saharan Africa and Uganda that have looked at similar issues. However, the presentation of the quantitative and qualitative findings could be improved to make a stronger case for the underlying story or narrative that the authors aim to share. Currently, the results part of the paper reads more like a report than an academic paper, with the data under-analyzed and presented for the reader to interpret. The qualitative data are basically presented as anecdotal quotes rather than in a way that is convincing evidence that this is the pattern that emerged – more description of difference (across sites, by age, by gender, diagnosis, etc) or highlighting outliers could strengthen the qualitative results.**

**Response:** Thank you for your thorough review of our manuscript and your valuable feedback on the results section. We have revised the entire results section to include more details that enhance the clarity of our findings. The updated results can now be found on ***lines 240-456, pages 14 to 28.***

**Comment 4: The tables are hard to read and interpret. For example, Figure 3 is not described in the text in a way that explains what these box plots mean. What is a ‘good’ KAP score? What is meaningfully missing when a KAP score is not good?**

**Response:** Thank you for pointing this out. We apologize for any confusion caused. To improve clarity, we have now removed Figure 3, as knowledge findings are already reported in Table 5. We appreciate your feedback in helping us enhance the readability of our tables.

**Comment 5: There is additional literature on aging, HIV, and NCDs in Uganda/SSA/LMICs that are not included in the references that might be useful – while it isn’t necessary to include all of them, it did seem like some key authors on Uganda were missing – Seeley, Wandera, etc. some of these are:**

**Coovadia, H., Jewkes, R., Barron, P., Sanders, D. and McIntyre, D. 2009. The health and health system of South Africa: historical roots of current public health challenges. The Lancet.**

**Droti, B. 2014. Availability of health care for older persons in primary care facilities in Uganda. PhD thesis, London School of Hygiene and Tropical Medicine, University of London, London.**

**Kuteesa, M. O., Seeley, J., Cumming, R. G. and Negin, J. 2012. Older people living with HIV in Uganda: Understanding their experience and needs. African Journal of AIDS Research.**

**Negin, J., Nyirenda, M., Seeley, J. and Mutevedsi, P. 2013. Inequality in health status among older adults in Africa: the surprising impact of anti-retroviral treatment. Journal of Cross-cultural Gerontology.**

**Nnko, S., Bukenya, D., Kavishe, B. B., Biraro, S., Peck, R., Kapiga, S., Grosskurth, H. and Seeley, J. 2015. Chronic diseases in North-West Tanzania and Southern Uganda. Public perceptions of terminologies, aetiologies, symptoms and preferred management. PLOS ONE**

**Rabkin, M., Kruk, M.E. and El-Sadr, W.M. 2012 HIV, aging and continuity care: strengthening health systems to support services for non-communicable diseases in low-income countries. AIDS**

**Schatz E, Seeley J, Negin J and Mugisha J. 2018. They “don’t cure old age”: Delays to health care access among older adults in rural Uganda. Ageing and Society.**

**Wandera, S.O., Kwagala, B. and Ntozi, J. 2015. Determinants of access to healthcare by older persons in Uganda: a cross-sectional study. International Journal for Equity in Health.**

**Response:** Thank you for these suggested readings. We have added a citation to one of these relevant articles in our revised manuscript.

**Comment 6: Minor issues**

**In Table 3 – says distance to the clinic – assume this is km, but it doesn’t specify**

**Response:** Thank you for this observation. The distance is indeed measured in kilometres, and we have now specified this in Table 3 for clarity.
