# Supplementary material for: Harnessing HIV clinics to deliver integrated hypertension care for People living with HIV in Uganda: A formative mixed methods study
Source: PLOS Glob Public Health. 2025 Jun 4;5(6):e0004701. doi: 10.1371/journal.pgph.0004701 (PMC12136366; doi:10.1371/journal.pgph.0004701)
Supplement: Attachment — Submitted filename: Re-reviewing for PLOS Global Public Health.docx [file pgph.0004701.s005.docx]

**Re-reviewing Uganda HIV/HTN manuscript for PLOS Global Public Health**

Thank you for your thorough response to my comments. Excellent work clarifying the integration models, the settings for clinical integration, and the differences between blood pressure outcomes by geography.

**General**: consider changing every instance of “patient” to “participant” to confirm with patient-centered language (<https://www.niaid.nih.gov/research/hiv-language-guide>).

**Introduction:**
Line 82: “However, in order to improve clinical outcomes (such as screening and blood pressure control), existing barriers to HTN and HIV integration must be addressed.” Screening is not a clinical outcome; would remove.

Line 110: “Prescription and dispensing of antihypertensive medications at the same pharmacy as antiretroviral therapy.” Not a complete sentence; please revise.

Line 112-119: I’m still a bit unclear on how your current study relates to PULESA- it is one of six studies within the larger PULESA NHLBI grant?

Line 356/Table 6: “Only 44% of patients reported having their had their blood pressure checked within the 357 last seven days with 24% being checked between eight and thirty days ago.” You mentioned in your response that ‘guidelines recommend two measurements per day for a minimum of three and ideally 7 days, with an average of the measurements being used to diagnose hypertension’ and that you used ‘BP checks within seven days as one proxy indicator for optimal care’. I do not see these recommendations in the [2020 Uganda HIV guidelines](https://www.prepwatch.org/wp-content/uploads/2023/08/Consolidated-Guidelines-for-the-Prevention-and-Treatment-of-HIV-and-AIDS-in-Uganda-2020.pdf), nor do I see anything there about home blood pressure monitoring. Can you please provide the guidance that recommends this? Can you please clarify in the text, if this is indeed correct, that your participants had home blood pressure cuffs and also please provide the frequency with which they were advised to check their home BPs? I am not convinced that “BP checks within seven days” should be considered a proxy indicator for optimal care in this setting - nor in any setting. Many HTN SOPs in subSaharan Africa call for [repeat BPs on a monthly basis](https://resolvetosavelives.org/wp-content/uploads/2023/06/MJAP-Protocol-Document-8-15.pdf) until HTN control is achieved.

Line 496: The sections on Relative Advantage and Costs seem to be making the same argument- consolidate?

Line 497: “Participants identified several advantages to the integration of HTN and HIV services, particularly in overcoming the barriers of fragmented care and **high costs associated with antihypertensive medications**.” I understand how service integration can decrease the cost of travel to reach the clinic when clients are making fewer visits, but how does integrated service delivery reduce the high costs of antiHTN medications? Nothing in the text supports this.

**Discussion:**

Line 648 - 674: I would make this section its own paragraph, and try to really tighten up the writing. There are a few themes here that are mentioned repeatedly and should be condensed (decreased transportation costs, inconsistent medication supply, increased convenience, improved adherence). I would remove the 3 mentions of ‘stigma’ here because it’s beyond this scope- I don’t think your research touched on integrated models reducing stigma.
