# Supplementary material for: Harnessing HIV clinics to deliver integrated hypertension care for People living with HIV in Uganda: A formative mixed methods study
Source: PLOS Glob Public Health. 2025 Jun 4;5(6):e0004701. doi: 10.1371/journal.pgph.0004701 (PMC12136366; doi:10.1371/journal.pgph.0004701)
Supplement: Attachment — Submitted filename: Response_to_Reviewers_06_March_2025.docx [file pgph.0004701.s007.docx]

Florence Ayebare

Infectious Diseases Research Collaboration (IDRC)

Plot 2C Nakasero Hill Road, Kampala, Uganda

Jepchirchir Kiplagat

Academic Editor

PLOS Global Public Health

6^th^ March 2025

**Re-submission of our Manuscript: Harnessing HIV clinics to deliver integrated hypertension care for People Living with HIV in Uganda: A formative mixed methods study.**

We thank the PLOS Global Public Health editorial team and reviewers for the opportunity to revise our manuscript further. The additional comments and suggestions have been helpful in improving it.

In the table below, we have provided a point-by-point response to editorial and reviewer comments. We have also indicated the line and page numbers where changes have been made for easy review.

Please get in touch with me if you need any additional information.

Florence Ayebare

Corresponding Author

ORCID ID: <https://orcid.org/0000-0002-3343-146X>

| **Additional Editor Comments Responses** | |
| --- | --- |
| 1. While attempts have been made to revise this manuscript, the authors still need to refine the discussion section to situate the findings within available literature in the field. | Thank you for this comment. We have revised the discussion section, and these changes are reflected on **lines 428-481, pages 25-28.** |
| **Reviewer 1** |  |
| Thank you for your thorough response to my comments. Excellent work clarifying the integration models, the settings for clinical integration, and the differences between blood pressure outcomes by geography. | We appreciate your valuable feedback on this. |
| General: Consider changing every instance of “patient” to ‘Participant” to confirm with patient-centered language (https://www.niaid.nih.gov/research/hiv-language-guide) | Thank you for your thoughtful suggestion. We have made the recommended changes, opting to use ‘**persons living with HIV’** (**PLHIV**) instead of ‘patient’ to align with patient-centered language. This terminology is consistent with recent publications in PLOS Global Public Health, and we have applied it consistently throughout the manuscript. |
| Introduction  Line 82: “However, in order to improve clinical outcomes (such as screening and blood pressure control), existing barriers to HTN and HIV integration must be addressed.” Screening is not a clinical outcome; would remove. | Thank you for this observation. **As suggested on line 86, page 6,** we have removed the reference to ‘screening’. |
| Line 110: “Prescription and dispensing of antihypertensive medications at the same pharmacy as antiretroviral therapy.” Not a complete sentence; please revise. | Thank you for this observation. We have removed this sentence since reference to ‘prescription and dispensation of antihypertensive medications and antiretroviral therapy at the same pharmacy’ has been made in previous paragraphs – **lines 109-114, pages 7-8.** |
| Line 112-119: I’m still a bit unclear on how your current study relates to PULESA – it is one of six studies within the larger PULESA NHLBI grant? | Thank you for seeking clarification. The PULESA Uganda study is one of six HLB SIMPLe Alliance projects funded by the NHLBI (www.hlbsimple.org). These six projects are being implemented in different countries: Uganda, Botswana, Nigeria, Zambia, South Africa, and Mozambique. The study we are reporting on was conducted during the formative phase of the Uganda project, known as “PULESA Uganda.” To clarify this connection, we have added a sentence and reference **on lines 120–121, page 8.** |
| Line 356/Table 6: “Only 44% of patients reported having had their blood pressure checked within the 7 last seven days with 24% being checked between eight and thirty days ago.” You mentioned in your response that ‘guidelines recommend two measurements per day for a minimum of three and ideally 7 days, with an average of the measurements being used to diagnose hypertension’ and that you used ‘BP checks within seven days as one proxy indicator for optimal care’. I do not see these recommendations in the 2020 Uganda HIV guidelines, nor do I see anything there about home blood pressure monitoring. Can you please provide the guidance that recommends this? Can you please clarify in the text, if this is indeed correct, that your participants had home blood pressure cuffs and also please provide the frequency with which they were advised to check their home BPs? I am not convinced that “BP checks within seven days” should be considered a proxy indicator for optimal care in this setting – nor in any setting. Many HTN SOPs in sub-Saharan Africa call for repeat BPs on a monthly basis until HTN control is achieved. | Thank you for seeking clarification on this finding. We aimed to assess whether participants had ever had their blood pressure measured, regardless of the setting—whether at a local clinic, health facility, or elsewhere—and to determine whether any monitoring was taking place. We did not intend to prescribe a specific frequency for blood pressure measurement but rather to capture self-reported experiences of blood pressure monitoring.  In our setting, persons living with HIV (PLHIV) typically do not have access to home blood pressure monitoring devices. As such, we expected that any reported blood pressure measurement would have been conducted at a public or private health facility.  You are correct that the 2020 Uganda HIV guidelines do not specify a required frequency for blood pressure monitoring. Our question about blood pressure checks within the past few days was intended purely for historical data collection rather than as an indicator of optimal care. **We have revised the paragraph on lines 328–330, page 20,** to clarify this point. |
| Line 496: The sections on Relative advantage and Costs seem to be making the same argument – consolidate? | Thank you for this suggestion. To streamline the argument, we have consolidated the sections on Relative Advantage and Costs. The revisions have been made on **lines 375–430, pages 24–26.** |
| Line 497: “Participants identified several advantages to the integration of HTN and HIV services particularly in overcoming the barriers of fragmented care and high costs associated with antihypertensive medications.” I understand how service integration can decrease the cost of travel to reach the clinic when clients are making fewer visits, but how does integrated service delivery reduce the high costs of antiHTN medications? Nothing in the text supports this | Thank you for this question. In Uganda, chronic conditions are covered by both public and private health facilities. While HIV and hypertension (HTN) services should, in principle, be provided at no direct cost in public facilities, frequent stockouts of antihypertensive medications often force patients to purchase them out-of-pocket from private pharmacies or drug shops. In contrast, antiretroviral therapy (ART) is consistently available at public health facilities. We propose an integrated service delivery model that could facilitate more reliable access to antihypertensive medications for persons living with HIV (PLHIV) at no cost, addressing the financial burden associated with purchasing these medications privately. |
| Discussion  Line 648 – 674: I would make this section its own paragraph, and try to really tighten up the writing. There are a few themes here that are mentioned repeatedly and should be condensed (decreased transportation costs, inconsistent medication supply, increased convenience, improved adherence). I would remove the 3 mentions of ‘stigma’ here because it’s beyond this scope – I don’t think your research touched on integrated models reducing stigma. | Thank you very much for this suggestion. We have revised this section and removed the references to stigma. The changes are reflected on **lines 456-479, page 27.** |
|  |  |
| **Reviewer 2** |  |
| I still find the qualitative aspects of this work under-analyzed. The authors made efforts to change this section – not just listing quotes – however, it still has too many sub-sections, not enough information about any variation across age/sex/geography/health status, and not enough information about how common particular answers were and what types of answers clustered together. | Thank you for your valuable feedback. We appreciate your comments on the qualitative analysis and have tried to address these concerns. In response, we have restructured the findings section using the Consolidated Framework for Implementation Research (CFIR) to provide a more systematic and comprehensive approach to the data. We’ve worked to refine the analysis by reducing unnecessary sub-sections, and we have added a more detailed exploration of variation across factors such as age, sex, geography, and health status, as well as insights into how these variations may influence responses. Additionally, we have provided further clarification on the frequency of specific answers and have grouped related responses to show meaningful patterns and clusters. We hope these changes address your concerns and enhance the depth of the analysis – **lines 246-427, pages 15-25.** |
| I also find the discussion to continue to be insufficient in engaging with extant literature on aging in Uganda, HIV and Chronic disease, and aging in under-resourced health care environments. | Thank you for your thoughtful feedback. We acknowledge that the discussion section could benefit from more engagement with the existing literature on aging in Uganda, HIV and chronic disease, and aging in under-resourced healthcare environments. In response to your comment, we have expanded our discussion to include a review of relevant literature, highlighting key studies and findings in these areas. These additions provide a stronger theoretical foundation and a more comprehensive context for our findings. We hope this revised section better addresses the gaps you identified. These changes are reflected on **lines 428-481, pages 25 -28.** |
| The paper has the potential to provide important information on the lives and experiences of older Ugandans living with HIV, so I hope that the authors continue to revise to make the paper as strong as it has the potential to be. | Thank you for your feedback. We greatly appreciate your recognition of the paper’s potential to contribute important insights into the experiences of older Ugandans living with HIV. We have revised the manuscript to ensure it fully captures these critical aspects. We focused on refining the presentation of the findings and deepening the discussion around the unique challenges faced by this population, particularly in relation to managing multiple chronic conditions. Your support and suggestions have been invaluable in guiding us towards making the paper stronger. |
